# Treatment dependent impact of plasma-derived exosomes from head and neck cancer patients on the epithelial-to-mesenchymal transition

**DOI:** 10.3389/fonc.2022.1043199

**Published:** 2023-01-04

**Authors:** Linda Hofmann, Marie Waizenegger, Ralph Röth, Stefanie Schmitteckert, Daphne Engelhardt, Patrick J. Schuler, Simon Laban, Thomas K. Hoffmann, Cornelia Brunner, Marie-Nicole Theodoraki

**Affiliations:** ^1^ Department of Otorhinolaryngology, Head and Neck Surgery, Ulm University Medical Centerr, Ulm, Germany; ^2^ nCounter Core Facility, Institute of Human Genetics, University of Heidelberg, Heidelberg, Germany

**Keywords:** exosomes, HNSCC, EMT, conventional therapy, recurrence

## Abstract

**Background:**

Epithelial to mesenchymal transition (EMT) is a key process in carcinogenesis of head and neck squamous cell carcinoma (HNSCC), contributing to tumor invasiveness, distant metastasis, and recurrence. Exosomes are known mediators and regulators of EMT. Here, we analyze the impact of exosomes that were primed by conventional therapy on EMT modulation.

**Methods:**

Plasmas of n = 22 HNSCC patients were collected before and after standard of care surgery and adjuvant or primary (chemo)radiotherapy. Exosomes were isolated by size exclusion chromatography. Upon co-incubation of exosomes with HNSCC cells, the cellular EMT profile was analyzed by flow cytometry and RT-qPCR. Wound healing assays were performed to evaluate migratory potential of exosome-treated cells.

**Results:**

Reduction of total exosome protein after therapy and *in vitro* exosome induced EMT profiles were dependent on the type of treatment. Exosomal TFG-β and miRNA cargo were partly responsible for observed exosome induced EMT changes. Exosomes from recurrent patients induced higher tumor cell migration after therapy than exosomes from disease-free patients.

**Conclusions:**

HNSCC patients’ exosomes from timepoints before and after therapy were able to confer therapy induced EMT modulation *in vitro* and have the potential to monitor the EMT process. Exosome induced changes in migratory potential emerged as discriminants of therapy outcome.

## Introduction

Head and neck squamous cell carcinoma (HNSCC) is one of the seven most common cancers worldwide (1). Despite multimodal treatment regimens consisting of surgery with adjuvant (chemo)radiotherapy (Sx + adj (C)RT) or primary chemoradiotherapy (prim CRT), treatment outcome remains poor (2). This is mainly attributed to recurrence and distant metastasis.

Epithelial to mesenchymal transition (EMT) is a key process in carcinogenesis and strongly contributes to tumor invasiveness and distant metastasis. During EMT, cancer cells progressively lose their epithelial phenotype and gain mesenchymal migratory properties (3). The cellular and molecular changes include loss of cell adhesion and cell polarity, reorganization of the cytoskeleton, decreased E-cadherin, and increased vimentin expression. EMT is accompanied and promoted by transforming growth factor ß (TGF-β) (4), several EMT-inducing transcription factors such as *TWIST*, *SNAIL* and *SLUG* (5) as well as regulatory miRNAs (6). EMT is further related to resistance to chemo- and immunotherapy (7).

Exosomes are increasingly attributed as mediators and regulators of EMT (8, 9) and their involvement in EMT progression has been shown for various tumor entities, such as lung (10), pancreatic (11), ovarian (12), esophageal (13), nasopharyngeal (14), and bladder (15) cancer. Exosomes are small extracellular vesicles which, upon release by the parental cells, mediate intercellular communication in both physiological and pathological settings (16). Due to their unique biogenesis in the endosomal compartment, their delivered cargo resembles the characteristics of the parental cell and consists of proteins, nucleic acids, and lipids (17, 18). Among the exosomal cargo contributing to EMT induction, TGF-β, HIF-1α and several miRNAs were identified (8, 19). We have previously shown that palliative therapeutic interventions change the exosomal cargo and their ability to induce EMT (20). Further, we recently identified changes in the molecular profile of exosomes from HNSCC patients treated with Sx + adj (C)RT which reflected response to therapy (21). However, the influence of these treatment regimens on exosome mediated EMT induction in HNSCC has not been investigated.

Here, we analyze the influence of conventional therapy (Sx + adj (C)RT or prim CRT) on the exosomal cargo and the ability of exosomes to modulate EMT. We evaluate whether plasma-derived exosomes reflect the degree of EMT, tumor aggressiveness, and therapy response in HNSCC.

## Material and methods

### Collection of blood samples

In this manuscript data from two HNSCC patient cohorts are included. For the main cohort, peripheral blood samples from 22 newly diagnosed HNSCC patients who were treated at the Department of ENT, Head and Neck Cancer Surgery at the University of Ulm were collected between 2015 and 2018. In accordance with international treatment guidelines, and by recommendation of the interdisciplinary tumor board at the Comprehensive Cancer Center Ulm, these patients were treated with surgery and adjuvant radiotherapy (Sx + adj RT), surgery and adjuvant chemoradiotherapy (Sx + adj CRT) or primary chemoradiotherapy (prim CRT). Blood was drawn at time of diagnosis (BT = before therapy) and after therapy (AT). The mean time between the end of therapy and blood draw was 86 ± 66 days. All experiments, except miRNA profiling, were conducted with exosomes from this patient cohort. For miRNA profiling, peripheral blood samples from another, treatment-naïve HNSCC patient cohort (n = 16) were collected. Additionally, blood from age- and sex-matched healthy donors (HD, n = 21) was collected. The mean age of the HD cohort was 58 years and 81% of HD were male. The collection of blood samples and clinical data of HNSCC patients and healthy donors were approved by the Ethics Committee at the University of Ulm (proposal # 90/15). Each patient provided informed consent. **Table 1** provides the clinicopathological characteristics of both HNSCC patient cohorts included in this study.

**Table 1:** Clinicopathological data of HNSCC patients enrolled in this study.

Characteristics	Patients main cohort (n = 22)		Patients miRNA profiling (n = 16)	
	n	%	n	%
**Age (years)**	Range 40-78 years		Range 52-79 years	
≤ 60	12	55	6	37.5
> 60	10	45	10	62.5
**Gender**				
Male	18	82	13	81
Female	4	18	3	19
**Primary tumor site**				
Oropharynx	11	50	7	44
Hypopharynx	1	4	4	25
Nasopharynx	1	4	1	6
Oral cavity	3	14	2	12.5
Larynx	3	14	2	12.5
Multiple sites	3	14		
**HPV status (only oropharynx)**				
Positive	5	45.5	2	28.5
Negative	5	45.5	3	43
Undefined	1	9	2	28.5
**Tumor stage**				
T1	0	0	2	12.5
T2	8	36	4	25
T3	6	28	4	25
T4	8	36	6	37.5
**Nodal status**				
N0	8	36	6	37.5
N+	14	64	10	62.5
**Distant metastasis**				
M0	22	100	16	100
**UICC grade**				
I/II (low)	5	23	2	12.5
III/IV (high)	17	77	14	87.5
**Therapy**				
Surgery + adjuvant RT	7	32		
Surgery + adjuvant CRT	5	23		
Primary CRT	10	45		
**Recurrence**				
Yes	6	27		
No	16	73		

HPV, Human papillomavirus; RT, Radiotherapy; CRT, Chemoradiotherapy; UICC, Union for international cancer control.

Blood samples were collected in citrate tubes and centrifuged at 1,000 x g for 10 min followed by 2,500 x g for 10 min. Resulting plasma was stored in aliquots at -20°C until further use.

Due to limited material and the unavailability of single corresponding samples, not all analyses could be performed with all samples. When analyzing paired samples BT and AT, samples with only one available timepoint were not considered.

### Exosome isolation by size exclusion chromatography exosome quantification and exosome concentration

Exosomes were isolated by size exclusion chromatography (SEC) as previously described (22). Briefly, plasma samples were thawed and centrifuged at 2,000 x g for 10 min and 10,000 x g for 30 min at 4°C. Then, plasma was filtered through 0.22 µm syringe-filters (Millipore, Burlington, MA, USA), loaded on SEC columns and eluted with 1x PBS. Sequential 1 mL fragments were collected, and fraction #4 is enriched in intact and biologically active exosomes.

Total exosomal protein (TEP) was quantified using Pierce BCA Protein Assay Kit (ThermoFisher Scientific, Waltham, MA, USA) according to the manufacturer’s protocol.

Exosomes were concentrated using 100 kDa cut-off centrifugal filter units (Millipore). For Western blot analysis, 20 µg of exosomes in 40 µL PBS were used and for co-incubation assays, 10 µg of exosomes in 100 µl were used, as described in the respective methods section.

### Characterization of exosomes

Exosomes were characterized using transmission electron microscopy, nanoparticle tracking, and Western Blot analysis. The methods are in line with the minimal information for studies of extracellular vesicles (MISEV) 2018 guidelines for the definition of extracellular vesicles (23) and are routinely performed as described in detail in our previous publication (24) (EV-TRACK ID: EV200068).

### Western blot of exosomes

For detection of exosomal TGF-β, 20 µg of exosomes in 40 µl PBS were lysed using Lane Marker Reducing Sample Buffer (ThermoFisher Scientific), heated at 95°C for 5 min and separated using 12% Mini-PROTEAN^®^ TGX™ SDS/PAGE Gels (BioRad, Hercules, CA, USA). Proteins were transferred to nitrocellulose membranes using the Transblot Turbo System (BioRad). Membranes were blocked with 5% BSA/TBS-T for 1 h at room temperature and incubated with TGF-ß antibody (RRID : AB_2063354, Cell Signaling Technology, Danvers, MA, USA) or TSG101 antibody (RRID : AB_2548734, ThermoFisher Scientific) over night at 4°C. The next day, membranes were washed three times with TBS-T and incubated with Goat anti-Rabbit IgG (H+L), HRP (RRID : AB_228341, Thermo Fisher Scientific) for 40 min at room temperature. Membranes were developed and analyzed with the Chemi Doc MP Imaging System and Image Lab 6.0.1 software (Biorad).

### Co-incubation of exosomes and tumor cell lines

The HNSCC cell line UD-SCC-1 (RRID : CVCL_E324, source Henning Bier, Düsseldorf, Germany) was cultured in DMEM (Gibco, Carlsbad, CA, USA) supplemented with 10% fetal bovine serum and 1% ZellShield (Minerva Biolabs, Berlin, Germany) at 37°C, 5% CO_2_ and > 90% relative humidity. The identity of the cell line was proven by short tandem repeats (STR) analysis and cultures were regularly checked for an absence of mycoplasma contamination. Cells grown to 60-70% confluency were used for experiments.

For co-incubation experiments, culture medium was changed to DMEM supplemented with 10% exosome-depleted FBS (Gibco) and 1% ZellShield. 200,000 cells in 500 µl medium were seeded in 48-well plates and allowed to attach for 24 h. Then, 10 µg exosomes in 100 µl PBS were added and incubated for 72 h. Downstream analysis comprised flow cytometry and RNA analysis as described in the following sections.

### Flow cytometry of surface protein levels

Cells were washed with PBS, detached using Trypsin/EDTA (PAN Biotech, Adenbach, Germany) and washed twice with PBS containing 2% BSA. Cells were stained with 5 µl FITC anti-human CD324 (E-cadherin) antibody (RRID : AB_756066, Biolegend, San Diego, CA, USA) or 5 µl FITC Mouse IgG1 κ Isotype Ctrl antibody (RRID : AB_2861401, Biolegend) for 30 min at 4°C in the dark. After two further washing steps, cells were measured with a Gallios flow cytometer (Beckman Coulter, Brea, CA, USA) and data were analyzed using Kaluza Analysis 2.1 software.

### Flow cytometry of intracellular protein levels

For intracellular measurements of vimentin, cells were prepared with BD Cytofix/Cytoperm (BD Biosciences) according to manufacturer’s protocol. Cells were stained with 5 µl Alexa Fluor 488 anti-human vimentin antibody (RRID : AB_10896994, BD Biosciences) or 5 µl Alexa Fluor 488 Mouse IgG1 κ Isotype Control (AB_396830, BD Biosciences) for 30 min at 4°C in the dark. After two washing steps, cells were measured with a Gallios flow cytometer and data were analyzed using Kaluza Analysis 2.1 software.

### RNA isolation and RT-qPCR

Cells were washed with PBS and harvested using 600 µl RLT-Buffer from the RNeasy Mini Kit (Qiagen, Hilden, Germany) supplemented with 2-mercaptoethanol (1:100). RNA was isolated according to manufacturer’s instruction. RNA concentrations were determined using a TECAN spectrophotometer (Männedorf, Switzerland).

cDNA was synthesized using the QuantiNova Reverse Transcription Kit (Qiagen) according to the manufacturer’s instruction. Briefly, 2 µL gDNA removal mix was mixed with 1,000 ng template RNA and RNase-free water, resulting in a final volume of 15 µL. The reaction mix was incubated for 2 min at 45°C in a thermocycler. Tubes were placed on ice and 4 µL of reverse transcription mix with 1 µL of reverse transcription enzyme were added. Reverse transcription was then performed in a thermocycler, with 3 min at 25°C, 10 min at 45°C, and 5 min at 85°C. cDNA was diluted 1:10 in RNase-free water.

RT-qPCR was performed using the QuantiNova SYBR Green PCR Kit (Qiagen). First, a reaction mix was prepared by mixing 10 µL of 2x SYBR Green PCR Master Mix, 0.6 µL forward primer, 0.6 µL reverse primer and 6.8 µl RNase-free water, resulting in a final volume of 18 µL. Then, 2 µL diluted cDNA was added, and RT-qPCR was run in a Roche Light Cycler 96 (Basel, Switzerland) using the following program: 2 min of initial heat activation at 95°C, followed by 2-step cycling of 5 s denaturation at 95°C and 10 s combined annealing and extension at 60°C, repeated for 40 cycles. All primers were manufactured by Biomers (Ulm, Germany) and are listed in **Supplementary Table S1**.

Experiments were performed in duplicate and with negative controls. Hypoxanthine phosphoribosyl transferase (*HPRT*) was used as the normalization control. The delta Ct value (ΔCt) was calculated between the target and the HPRT mean of the same condition. Relative target mRNA expression levels compared to HPRT were calculated as 2^-ΔCt^.

### Wound healing assay

For wound healing assays, 400,000 cells in 1 ml medium were seeded in 24-well plates and allowed to attach for 24 h. 10 µg exosomes in 100 µl PBS were added and incubated for 24 h. Then, the cell monolayer was scratched using a 10 µl pipette tip and documented using an Olympus CK30 microscope (Zeiss, Oberkochen, Germany) at 5-fold magnification. Cells were incubated for another 24 h and the scratch was photographed again. Analysis was performed using ImageJ software (RRID : SCR_003070) by determining the wound area at 0 h (equals 100% wound area) and 24 h after the injury. The closed wound area (in %) was calculated as 100% - (wound area 24 h after injury/wound area 0 h after injury * 100%).

### Exosomal miRNA profiling

Exosomal miRNA profiling was performed with samples from the second, treatment-naïve patient cohort. Total exosomal RNA was isolated using the miRNeasy Micro Kit (Qiagen) according to the manufacturer’s instructions. Quality and amount of exosomal RNA were assessed by Agilent 2100 Bioanalyzer (Agilent Technologies) and a Qubit Fluorometer (Thermo Fisher Scientific). Exosomal miRNA profiling was performed using the nCounter^®^ SPRINT system (Nanostring Technologies) at the nCounter^®^ Core Facility of the University of Heidelberg, Germany. Total exosomal RNA was applied to the Human v3 miRNA Assay (CSO-MIR3-12, Nanostring Technologies) covering the measurement of 827 human miRNAs. A gene list providing accession numbers and target sequences can be downloaded from the company homepage. The assay was performed with 500 pg of total exosomal RNA according to the manufacturer’s instructions.

miRNA data was analyzed using the nSolver 4.0 software. Raw counts of individual miRNAs were normalized using the positive ligation controls. Differential presence of miRNAs between HNSCC and HD exosomes was calculated by Mann-Whitney test. Using TargetScanHuman 8.0, all miRNAs from the database were selected, which were predicted to target *CDH1, VIM, TWIST1* and *SNAI1/2*, and compared to miRNAs identified in our cohort. Counts of intersecting miRNAs were compared between exosomes of HNSCC patients and HD. Functional analysis of this miRNA set was performed using the miRNA Enrichment Analysis and Annotation Tool (miEAA).

### Data deposition and statistical analysis

Flow cytometry raw data (fcs files) is available at Flow Repository (flowrepository.org), accession number FR-FCM-Z5R6.

Statistical analysis was performed using GraphPad Prism (version 9, GraphPad Software, San Diego, CA, RRID : SCR_002798). Data is indicated as before-after blots or box-and-whisker blots with the line representing the median, the box showing the interquartile range (25-75%) and the whiskers indicating the range. Wilcoxon test was used to compare related samples and Mann-Whitney test was used to compare independent samples. *P* < 0.05 was considered statistically significant. Pearson’s correlation analysis was used to identify correlations between single outcomes.

## Results

### Study population

Clinicopathological characteristics of the HNSCC patients involved in this study are listed in **Table 1**.

At time-point of diagnosis, the mean age of the main cohort was 58 years with a range from 40 to 78 years. The majority of patients was male (82%). The primary tumor was located in the pharynx (58%), oral cavity (14%), larynx (14%), or in multiple sites (14%). Among patients with an oropharyngeal tumor (n = 11), the human papillomavirus (HPV) status, routinely determined by p16 immunohistochemistry and PCR for HPV-DNA, was positive in 5 patients, negative in 5 patients, and not evaluated in 1 patient. 64% of patients presented with an advanced tumor stage (T3/4) and the same amount had lymph node metastasis. According to the 8^th^ edition of Union for International Cancer Control (UICC), 23% percent of the patients were UICC stage I or II, and 77% were UICC stage III or IV. 7 patients (32%) were treated with Sx + adj RT, 5 (23%) with Sx + adj CRT and 10 (45%) with prim CRT. 6 of 22 patients (27%) developed recurrence or showed progressive or residual disease after therapy.

The mean age of the cohort used for miRNA profiling was 64 years with a range from 52 to 79 years and 81% of patients were male. The primary tumor was located in the pharynx (75%), oral cavity (12.5%), or larynx (12.5%). Among the patients with an oropharyngeal tumor (n = 7), HPV status was positive in 2 patients, negative in 3 patients, and not evaluated in 2 patients. 62.5% of patients presented with an advanced tumor stage (T3/4) and the same amount had lymph node metastasis.

### Characterization of plasma-derived exosomes

Plasma-derived exosomes were evaluated for morphology, size, and protein composition (**Supplementary Figure S1**). They showed typical vesicular morphology and were within the normal range of exosome size with a median diameter of around 80 nm. Exosome preparations contained the vesicle associated proteins CD9 and CD63 as well as the endosomal marker TSG101, while the cellular marker Grp94 and apolipoprotein ApoA1 were not detected.

### TEP is reduced after therapy depending on type of treatment

**Figure 1:**
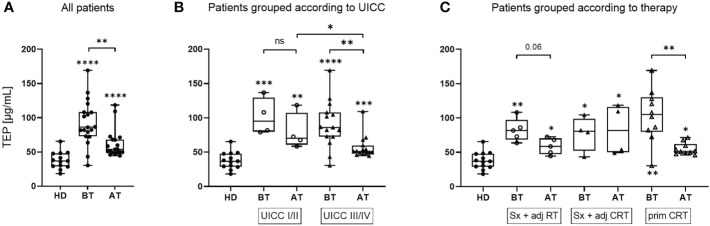
TEP values of exosomes isolated from plasma of HNSCC patients before and after therapy. Total exosome protein (TEP) concentrations of the exosome fraction obtained from plasma of n = 13 healthy donors (HD) and n = 19 head and neck squamous cell carcinoma (HNSCC) patients before therapy (BT) and after therapy (AT). Results are shown ungrouped **(A)** or grouped according to Union for International Cancer Control (UICC) stage **(B)** or therapy regiment **(C)**, as indicated in the text boxes. N = 4 HNSCC patients were UICC I/II and n = 15 were UICC III/IV. N = 5 HNSCC patients were treated with surgery + adjuvant radiotherapy (Sx + adj RT), n = 4 with surgery and adjuvant chemoradiotherapy (Sx + adj CRT) and n = 10 with primary chemoradiotherapy (prim CRT). Results are plotted as box-and-whisker blots representing the median value, the 25th and 75th quartiles and the range. Wilcoxon related samples tests were applied to compare BT and AT. Mann-Whitney test was applied to compare HD with each BT or AT. *, **, *** and **** correspond to *p* ≤ 0.05, *p* ≤ 0.01, *p* ≤ 0.001 and *p* ≤ 0.0001, respectively. ns, not significant. Asterisks above boxes indicate the significance compared to HD.

TEP was measured as an estimate of the exosome load. Compared to HD, TEP was significantly elevated in HNSCC patients both before and after therapy (**Figure 1A**). However, patients showed significantly reduced TEP amounts after therapy compared to before therapy. This decrease of TEP was especially profound in UICC high grade patients (**Figure 1B**). After therapy, UICC high grade patients had significantly lower TEP compared to UICC low grade patients. Furthermore, a reduction of TEP after therapy was only visible in patients treated with Sx + adj RT or prim CRT, while patients treated with Sx + adj CRT did not show relevant changes (**Figure 1C**).

### Exosomes influence the epithelial and mesenchymal protein profile of tumor cells *in vitro*

**Figure 2:**
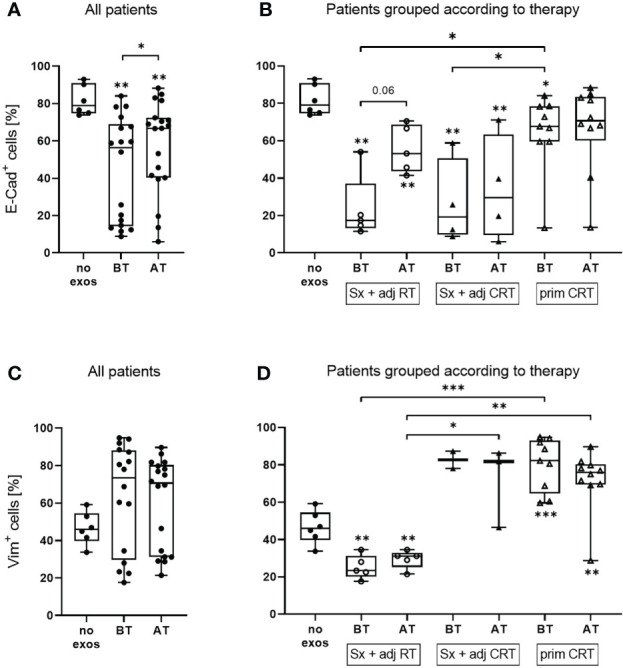
E-cadherin and vimentin protein expression of UD-1 cells upon co-incubation with exosomes isolated from plasma of HNSCC patients before and after therapy. UD-1 cells were co-incubated for 72 h with exosomes isolated from plasma of n = 19 HNSCC patients BT and AT. Following, E-cadherin **(A, B)** and vimentin **(C, D)** expression of co-incubated UD-1 cells was assessed by flow cytometry. As controls, UD-1 cells without exosome treatment (no exos, n = 6) were included. Results are shown ungrouped **(A, C)** or grouped according to therapy **(B, D)**. N = 5 HNSCC patients were treated with Sx + adj RT, n = 4 with Sx + adj CRT and n = 10 with prim CRT. Results are plotted as box-and-whisker blots representing the median value, the 25th and 75th quartiles and the range. Wilcoxon related samples tests were applied to compare BT and AT. Mann-Whitney test was applied to compare no exos with each BT or AT. *, ** and *** correspond to *p* ≤ 0.05, *p* ≤ 0.01 and *p* ≤ 0.001, respectively. Asterisks above boxes indicate the significance compared to no exos.

Flow cytometry measurement of E-cadherin and Vimentin levels was used to assess the *in vitro* EMT profile of tumor cells in a quantitative and sensitive manner. Under basal conditions, UD-SCC-1 (UD-1) cells showed a rather epithelial profile with ~80% E-cadherin positive and ~45% vimentin positive cells (**Figures 2A, C**). To evaluate exosome induced changes in the protein profile of tumor cells *in vitro*, UD-1 cells were co-incubated with exosomes from patients before and after therapy and their expression of E-cadherin and vimentin was measured by flow cytometry. Cells treated with exosomes from both timepoints showed a significantly reduced E-cadherin expression compared to untreated cells (**Figure 2A**). However, exosomes from patients after therapy induced a significantly lower E-cadherin inhibition compared to exosomes from patients before therapy. When grouping the patients according to therapy, exosomes from patients treated with Sx + adj RT or Sx + adj CRT led to a strong reduction of E-cadherin expression before therapy (**Figure 2B**). Only in patients treated with Sx + adj RT the E-cadherin expression was less suppressed with exosomes after therapy, while in patients treated with Sx + adj CRT there was no change in E-cadherin expression after therapy. Compared to the other therapy groups, exosomes from patients treated with prim CRT showed only a minimal decrease of E-cadherin expression before therapy and did not change after therapy.

UD-1 cells co-incubated with exosomes from patients before and after therapy showed an increase of vimentin expression compared to untreated cells, however it was not significant due to the great variability of the single patients (**Figure 2C**). Exosomes from patients treated with Sx + adj RT caused a reduced vimentin expression while patients treated with Sx + adj CRT or prim CRT caused an increased Vimentin expression compared to the untreated control (**Figure 2D**). Vimentin induction with exosomes from patients treated with Sx + adj RT was significantly lower compared to the other therapy groups. No difference in vimentin expression with exosomes from the timepoint before and after therapy was observed for any of the therapy groups.

### Exosomes influence the mRNA expression of EMT markers and transcription factors of tumor cells *in vitro*

**Figure 3:**
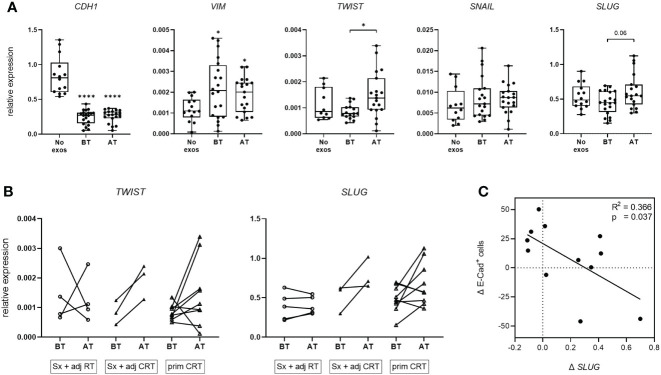
mRNA expression of EMT markers and transcription factors of UD-1 cells upon co-incubation with exosomes isolated from plasma of HNSCC patients. **(A, B)** UD-1 cells were co-incubated with exosomes isolated from plasma of n = 22 HNSCC patients BT and AT. mRNA expression of E-cadherin, vimentin and the transcription factors *TWIST*, *SNAIL* and *SLUG* was analyzed by RT-qPCR and normalized to *HPRT* using 2^-ΔCt^ algorithm. Target mRNA levels are shown as relative expression to *HPRT*. As controls, UD-1 cells without exosome treatment (no exos, n = 14) were included. In **(A)**, results are shown ungrouped and are plotted as box-and-whisker blots representing the median value, the 25th and 75th quartiles and the range. Wilcoxon related samples tests were applied to compare BT and AT. Mann-Whitney test was applied to compare no exos with each BT or AT. * and **** correspond to *p* ≤ 0.05 and *p* ≤ 0.0001, respectively. Asterisks above boxes indicate the significance compared to no exos. In **(B)**, results are grouped according to therapy and shown as before-after blots. N = 4 HNSCC patients were treated with Sx + adj RT, n = 3 with Sx + adj CRT and n = 9 with prim CRT. **(C)** Pearson correlation analysis of n = 12 HNSCC patients between the exosome induced change of *SLUG* mRNA expression (Δ *SLUG*) and E-cadherin protein expression (Δ E-Cad^+^ cells) in UD-1 cells, calculated as the difference between BT and AT (expression_AT_ - expression_BT_).

To evaluate exosome induced changes in the mRNA profile of tumor cells *in vitro*, UD-1 cells were co-incubated with exosomes from patients before and after therapy and their expression of E-cadherin and vimentin as well as the EMT-related transcription factors *TWIST*, *SNAIL* and *SLUG* was measured by RT-qPCR. Cells co-incubated with exosomes from patients before and after therapy showed significantly reduced E-cadherin mRNA expression compared to untreated cells with no difference between before and after therapy (**Figure 3A**). Vimentin expression was significantly elevated compared to untreated cells, with no difference between the timepoints. For the transcription factors *TWIST*, *SNAIL* and *SLUG*, no differential expression between untreated and exosome-treated cells was observed. However, exosomes from patients after therapy induced significantly higher *TWIST* expression compared to exosomes from patients before therapy. The same trend was observed for *SLUG* expression (p = 0.06). This exosome induced increase of *TWIST* and *SLUG* expression after therapy was observed for most patients treated with Sx + adj CRT or prim CRT, but barley for patients treated with Sx + adj RT (**Figure 3B**). Pearson’s correlation analysis revealed a significant negative correlation between changes of *SLUG* expression and change of E-cadherin induction on protein level (**Figure 3C**).

### Exosomal TFG-β and miRNA cargo are partly responsible for the exosome induced EMT changes *in vitro*

**Figure 4:**
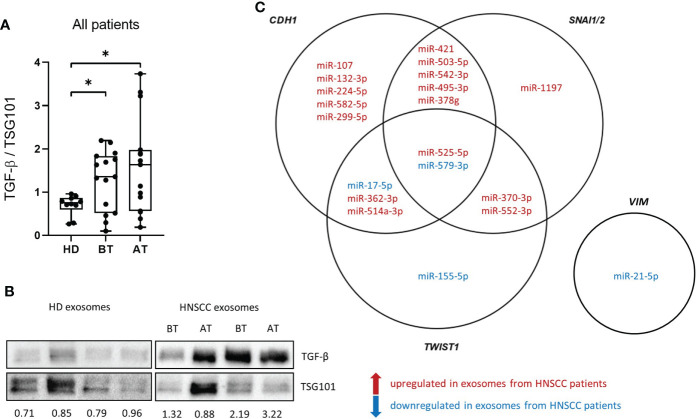
Transforming growth factor ß (TGF-ß) levels and miRNA cargo of exosomes isolated from plasma of HNSCC patients. **(A)** TGF-ß levels of exosome isolated from plasma of n = 10 HD and n = 15 HNSCC patients BT and AT were measured by western blot and normalized to the exosomal marker protein TSG101. Results are plotted as box-and-whisker blots representing the median value, the 25th and 75th quartiles and the range. Mann-Whitney test was applied to compare HD with each BT or AT. * corresponds to *p* ≤ 0.05. **(B)** Representative western blot images of exosomes analyzed for TGF-β and TSG101. Numbers below blots indicate relative exosomal TGF-β intensity normalized to TSG101. **(C)** Venn diagram of exosomal miRNAs targeting *CDH1*, *VIM*, *TWIST1* and *SNAI1/2*. miRNAs in red are upregulated in exosomes from HNSCC patients compared to exosomes from HD and miRNAs in blue are downregulated in HNSCC compared to HD exosomes.

Western blot analysis of exosomes isolated from patients before and after therapy was performed for TGF-β detection as one possible driver of the observed exosome induced EMT changes (**Figures 4A, B**). Patient exosomes showed significantly higher levels of TGF-β compared to HD exosomes (**Figure 4A**). However, no change was observed between exosomes from different timepoints. As miRNAs are potent influencers of EMT processes as well, the presence of exosomal miRNAs targeting the EMT-related genes *CDH1* (encoding E-cadherin), *VIM*, *TWIST1* and *SNAI1/2* (representing *SLUG* and *SNAIL*, respectively) was analyzed in treatment-naïve exosomes from HNSCC patients. 20 exosomal miRNAs targeting these 5 genes were extracted (**Figure 4C**). 13 out of 15 miRNAs (87%) targeting *CDH1* were upregulated in exosomes from HNSCC patients compared to HD (**Supplementary Figure 2**), which is consistent with the reduced expression of E-cadherin protein and mRNA observed with exosomes from before therapy *in vitro*. One miRNA targeting *VIM* was identified and found to be downregulated in exosomes from HNSCC patients compared to HD (**Supplementary Figure 2**), which fits the observation of higher vimentin protein and mRNA levels with exosomes from before therapy *in vitro*. 5 out of 8 (63%) upregulated exosomal miRNAs in HNSCC patients’ exosomes target *TWIST1* and 10 out of 11 (91%) target *SNAI1/2*. Although the majority of exosomal miRNAs targeting these transcription factor genes were upregulated in exosomes from HNSCC patients compared to HD (**Supplementary Figure 2**), no effect was observed with exosomes from before therapy *in vitro*, indicating an absence of translational control under these settings or the presence of compensatory mechanism.

### Disease dependent changes in the migratory potential of tumor cells incubated with exosomes *in vitro*

**Figure 5:**
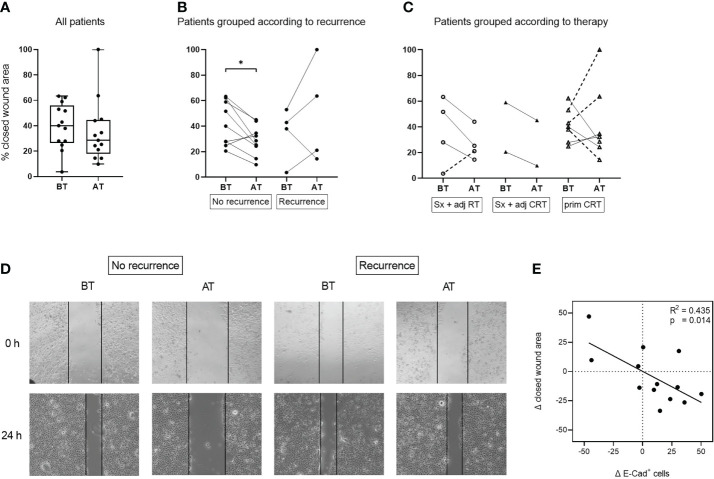
Migratory potential of UD-1 cells after co-incubation with exosomes isolated from plasma of HNSCC patients before and after therapy. UD-1 cells were co-incubated with exosomes isolated from plasma of n = 13 HNSCC patients BT and AT. After the first 24 h of co-incubation, cells were injured with a scratch and incubated for another 24 h Results are shown as % of closed wound area. Ungrouped results **(A)** are plotted as box-and-whisker blots representing the median value, the 25th and 75th quartiles and the range. Results grouped according to recurrence **(B)** or therapy **(C)** are plotted as before-after blots. N = 9 HNSCC patients had no recurrence, while n = 4 had a recurrence. N = 4 HNSCC patients were treated with Sx + adj RT, n = 2 with Sx + adj CRT and n = 7 with prim CRT. Wilcoxon related samples tests were applied to compare BT and AT. * corresponds to *p* ≤ 0.05. **(D)** Representative images of UD-1 cells (5-fold magnification) co-incubated with exosomes from plasma of a non-recurrent and a recurrent HNSCC patient before and after therapy at 0 h and 24 h after the scratch. **(E)** Pearson correlation analysis of n = 13 HNSCC patients between the exosome induced change of E-cadherin protein expression (Δ E-Cad^+^ cells) and migratory potential (Δ closed wound area) of UD-1 cells, calculated as the difference between BT and AT (expression_AT_ - expression_BT_; closed wound area_AT_ – closed wound area_BT_).

The migratory potential of UD-1 cells co-incubated with exosomes from patients before and after therapy was evaluated using a wound healing assay. Exosomes from patients after therapy induced a lower migratory potential compared to exosomes from patients before therapy, although this difference was not significant (**Figure 5A**). The decrease of migratory potential with exosomes from patients after therapy was only visible in patients who remained disease free after therapy (**Figures 5B, D**). In contrast, exosomes from patients with recurrence induced a higher tumor cell migration after therapy in 3 of 4 cases. The exosome induced change in migratory potential was not significantly influenced by the different therapy regiments (**Figure 5C**). Pearson’s correlation analysis revealed a significant negative correlation between change of E-cadherin protein expression and migratory potential: The higher the E-cadherin induction with exosomes after therapy, the lower the migratory potential with the same exosomes (**Figure 5E**).

## Discussion

In this study we analyzed the treatment dependent impact of exosomes from HNSCC patients undergoing conventional therapies (Sx + adj(C)RT or prim CRT) on EMT modulation. We evaluated whether plasma-derived exosomes reflect therapy induced changes in EMT, tumor aggressiveness, and therapy response in HNSCC.

Therapy induced changes were observed for TEP, *in vitro* EMT induction and migratory potential, but not exosomal TGF-β levels. TEP, as known indicator for exosomal load and disease activity, was significantly elevated in HNSCC patients before therapy compared to HD which is in line with previous studies (25, 26). We reported earlier that TEP is an indicator for response to therapy in HNSCC patients treated with PDT (20) or Sx + adj(C)RT (21). Likewise, TEP levels were significantly reduced in exosomes from patients after therapy in the present study, although they did not reach TEP levels of HD. The decrease in TEP was mainly attributed to patients treated with Sx + adj RT or with prim CRT, but not to patients treated with Sx + adj CRT. This is in line with previous data showing that exosome levels after surgery with adjuvant CRT were higher as compared to surgery alone (25). Reduced TEP in patients with Sx + adj RT and prim CRT might indicate reduced tumor load and the unaltered TEP in patients with Sx + adj CRT could be explained by the highest stimulation of the immune system by this therapy regimen and/or the lowest patient number in this group. Both tumor- (TEX) and immune cell-derived exosomes contribute to the total exosome population, and thus TEP, in plasma. Separation of exosome subpopulations showed that TEX are reduced after surgery, while CD3+ T cell derived exosomes and overall TEP levels are increased (21). Compared to surgery and RT or primary CRT, the combination of surgery and CRT might lead to the strongest activation of the immune system with an elevated release of immune cell-derived exosomes, which can mask a reduction of TEX. Elevated exosome levels during chemotherapy were also reported for hepatocellular and breast cancer, probably due to drug induced cellular stress (27–29). It will be necessary to discriminate exosomes subgroups to identify their contributions to the total exosome population.

Treatment-naïve exosomes from HNSCC patients were able to induce EMT *in vitro* with loss of E-cadherin expression and gain of vimentin expression, as it was reported for hepatocellular carcinoma (30, 31) or lung cancer (10). On protein level, the ability to induce EMT was reduced with exosomes from HNSCC patients after conventional therapy. However, there were strong differences in exosome induced EMT-modulation dependent on the therapy regimen. Before therapy, exosomes from patients treated with surgery and adjuvant RT induced both lower E-cadherin and vimentin expression. After therapy, they induced an EMT profile which is more favorable for the patient with increased E-cadherin and constant vimentin expression compared to the timepoint before therapy. This is in line with reduced TEP after therapy and generally resembles a low aggressive exosome profile in these patients. In contrast, both exosomes before and after therapy from patients treated with surgery and adjuvant CRT induced a highly mesenchymal profile in recipient tumor cells (classical EMT with reduced E-cadherin and increased vimentin expression) with no difference between the timepoints. At the same time, TEP did not change before and after therapy in this treatment regimen. One reason for the still highly aggressive exosome profile of these patients after therapy might be that tumor cells surviving surgery and CRT are more aggressive compared to the other treatment regimens. Similarly, exosomes from patients treated with primary CRT did not show differences in EMT-modulation before and after therapy. At both timepoints, these exosomes moderately induced EMT with simultaneously high E-cadherin and vimentin expression. This modulation could be attributed to an intermediate type of EMT, known as partial EMT, in which malignant cells adopt mesenchymal markers while they retain epithelial characteristics (32).

Sampling timepoints and treatment regimens caused differential exosome induced EMT modulation on protein level, while they did not influence mRNA levels of E-cadherin or vimentin. Still, the EMT-inducing transcription factors *TWIST* and *SLUG* were elevated after therapy compared to before therapy, notably only in patients receiving therapy with chemotherapy component. Higher mRNA levels of EMT-inducing transcription factors after therapy with a simultaneously more epithelial phenotype and less migration can be explained by the prevalence of partial EMT or (post-) transcriptional gene silencing through miRNAs. Indeed, *SLUG* was shown to correlate with partial EMT and its promotion (33).

Although the migratory potential of tumor cells did not significantly change between timepoints (before vs after therapy), it was associated with therapy outcome: only exosomes from non-recurrent patients induced significantly lower migration after therapy, while exosomes from patients with recurrence did not, or even convey, increased migratory potential.

We investigated exosomal TGF-β and miRNAs as mediators of EMT and confirmed that TGF-β is part of the exosomal cargo in HNSCC as described previously (34, 35). Further, we identified 20 exosomal miRNAs which were differentially present in exosomes from HNSCC patients compared to healthy exosomes and which are predicted to target EMT-related genes (*CDH1, VIM, TWIST1, SNAI1/2*). *In silico* analysis revealed their annotation to GO terms “cell proliferation” (95%), “TGF-β receptor signaling pathway” (80%) “positive regulation of epithelial cell migration” (70%) and positive regulation of EMT” (70%). Thus, both exosomal TGF-β and miRNAs can contribute to treatment-naïve, exosome induced EMT. However, there was no association of exosomal TGF-β levels with sampling timepoint (before vs after therapy) or treatment regimen, indicating that exosomal TGF-β was not mainly responsible for the treatment dependent changes in EMT modulation. It remains unclear, if miRNAs or other components of the exosomal cargo are responsible for treatment dependent changes in EMT modulation, as we were not able to analyze the exosomal miRNA cargo after therapy due to limited sample material. Nevertheless, studies described that the exosomal miRNA profile was altered upon EMT in lung cancer (36). Changes in the miRNA cargo of exosomes from HNSCC patients before and after therapy are most likely and need to be investigated in a separate patient cohort. Given the fact, that exosomal miRNAs are involved in EMT modulation, another interesting approach for the future is the possibility of preventing metastasis, thereby contributing to HNSCC treatment, by targeting exosomal miRNAs with oncogenic properties.

We are aware of several limitations of our study. First, the relatively small sample size of HNSCC patients and the variability among individuals limit the evidence and larger cohorts are needed to verify the observed effects on EMT modulation. Second, the timing of blood draw after treatment varies widely, as such we cannot completely rule out time-dependent effects. However, most importantly, all blood draws were taken before clinical evidence of recurrence or progressive disease. Third, miRNA data only shows an association of exosomal miRNAs with EMT modulation but does not prove a direct regulatory role on the target genes. Previous reports validated the transcriptional regulation of *SNAIL* and *TWIST1* by miR-370-3p in hepatocellular carcinoma, thereby suppressing metastasis (37), as well as *CDH1* by miR-421 in HNSCC, thereby promoting cell invasion and proliferation (38). Another study implies a role of miR-224 in development and progression of hepatocellular carcinoma by binding to target genes such as *CDH1* (39). Further validation experiments are required to address regulatory mechanisms of EMT modulation by exosomal miRNAs in HNSCC.

Overall, TEP and exosome induced changes of *in vitro* EMT profiles were different after therapy and dependent on the treatment combination. The surgery component mainly influenced exosome induced EMT modulation through an unfavorable *epithelial* profile (decreased E-cadherin protein), while the chemotherapy component mainly caused an unfavorable, aggressive *mesenchymal* profile (increased vimentin protein and *TWIST/SLUG* mRNA). Further, exosome induced changes in migratory potential emerged as discriminants of therapy outcome. As such, exosomes from plasma of HNSCC patients undergoing conventional treatment were able to recapitulate the therapy induced EMT modulation and have the potential to monitor the EMT process.

## Data availability statement

The data presented in the study are deposited in the Flow repository (https://flowrepository.org/id/FR-FCM-Z5R6) accession number FR-FCM-Z5R6 and ArrayExpress (https://www.ebi.ac.uk/biostudies/studies/E-MTAB-12408), accession number E-MTAB-12408.

## Ethics statement

The studies involving human participants were reviewed and approved by Ethics Committee of Ulm University. The patients/participants provided their written informed consent to participate in this study.

## Author contributions

LH, MW and RR performed experiments and analyzed the data. LH wrote the manuscript. MNT designed and supervised the study and helped drafting the manuscript. MNT, TKH, CB and SS provided resources. DE, PJS, SL, TKH and CB provided advice and reviewed/edited the manuscript. All authors contributed, read and agreed to the submitted version of the manuscript.

## Funding

This work was supported in part by the German Research Foundation (DFG) to MNT under Grant TH 2172/2–1.

## Conflict of interest

The authors declare that the research was conducted in the absence of any commercial or financial relationships that could be construed as a potential conflict of interest.

## Publisher’s note

All claims expressed in this article are solely those of the authors and do not necessarily represent those of their affiliated organizations, or those of the publisher, the editors and the reviewers. Any product that may be evaluated in this article, or claim that may be made by its manufacturer, is not guaranteed or endorsed by the publisher.
