# Supplementary material for: Treatment dependent impact of plasma-derived exosomes from head and neck cancer patients on the epithelial-to-mesenchymal transition
Source: Front Oncol. 2023 Jan 4;12:1043199. doi: 10.3389/fonc.2022.1043199 (PMC9845705; doi:10.3389/fonc.2022.1043199)
Supplement: Supplementary file 1 [file DataSheet_1.pdf]

Supplementary Material

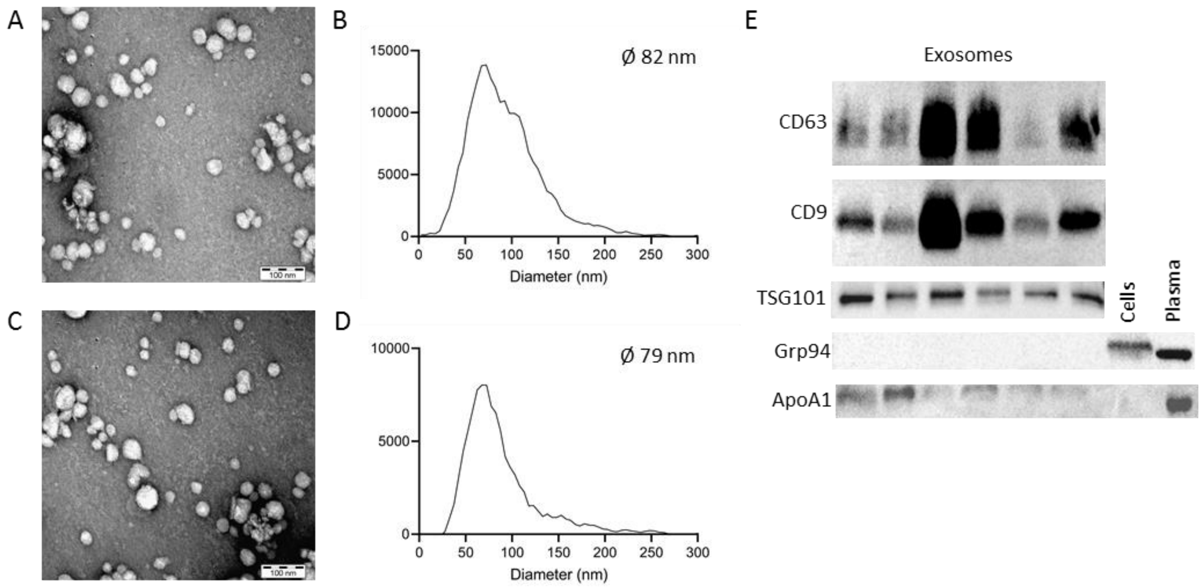

Supplementary Figure S1: Characterization of exosomes isolated from plasma. (**A, C**) Representative transmission electron microscopy (TEM) image of exosomes. Scalebar = 100 nm. (**B, D**) Representative size distribution of exosomes measured by nanoparticle tracking analysis (NTA). (**A, B**) are samples from before therapy and (**C, D**) are samples from after therapy. (**E**) Exosomes isolated from plasma of HNSCC patients were analyzed by Western blot for the presence of exosome specific markers using antibodies against CD63, CD9, TSG101, Grp94 and ApoA1. PCI-13 cell lysate and plasma were used as positive controls for Grp94 and ApoA1, respectively.

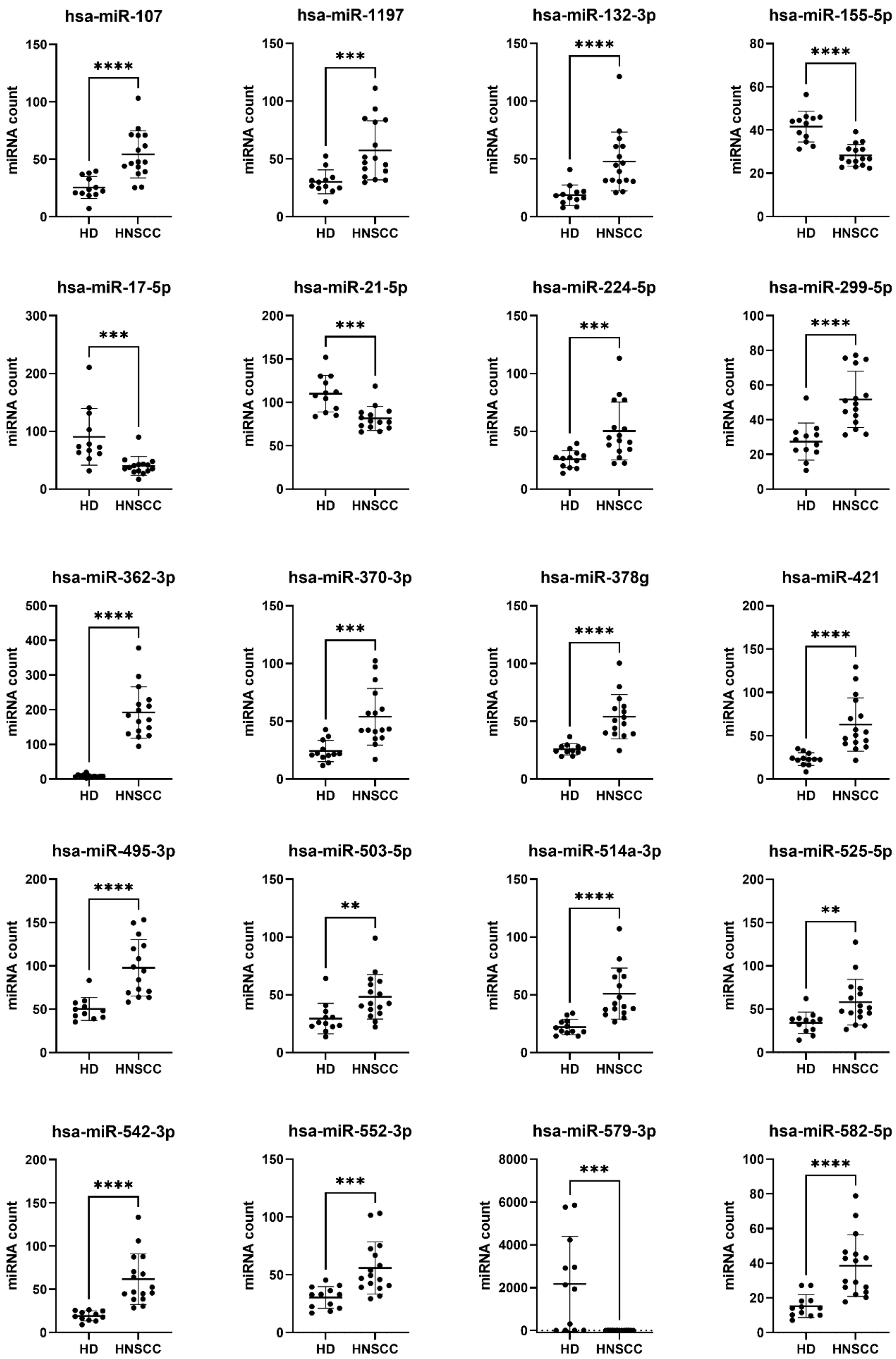

Supplementary Figure S2: Normalized counts of EMT-related miRNAs. Differential expression between HD and HNSCC was determined by Mann-Whitney test. **, ***, **** correspond to $p \leq 0.01$, $p \leq 0.001$ and $p \leq 0.0001$, respectively.

Supplementary Table S1: RT-qPCR primers

**Primer name**	**Primer sequence**
E-Cadherin forward	5’-CGAGAGCTACACGTTCACGG-3’
E-Cadherin reverse	5’-GGGTGTCGAGGGAAAAATAGG-3’
Vimentin forward	5’-GAGAACTTTGCCGTTGAAGC-3’
Vimentin reverse	5’-GCTTCCTGTAGGTGGCAATC-3’
Twist forward	5’-GGAGTCCGCAGTCTTACGAG-3’
Twist reverse	5’-TCTGGAGGACCTGGTAGAGG-3’
Snail forward	5’-TCGGAAGCCTAACTACAGCGA-3’
Snail reverse	5’-AGATGAGCATTGGCAGCGAG-3’
Slug forward	5’-AGATGCATATTCGGACCCAC-3’
Slug reverse	5’-CCTCATGTTTGTGCAGGAGA-3’
HPRT forward	5'- GACCAGTCAACAGGGGACAT-3’
HPRT reverse	5'- GTGTCAATTATATCTTCCACAATCAAG-3’
